# *MedShapeNet* – a large-scale dataset of 3D medical shapes for computer vision

**DOI:** 10.1515/bmt-2024-0396

**Published:** 2024-12-30

**Authors:** Jianning Li, Zongwei Zhou, Jiancheng Yang, Antonio Pepe, Christina Gsaxner, Gijs Luijten, Chongyu Qu, Tiezheng Zhang, Xiaoxi Chen, Wenxuan Li, Marek Wodzinski, Paul Friedrich, Kangxian Xie, Yuan Jin, Narmada Ambigapathy, Enrico Nasca, Naida Solak, Gian Marco Melito, Viet Duc Vu, Afaque R. Memon, Christopher Schlachta, Sandrine De Ribaupierre, Rajnikant Patel, Roy Eagleson, Xiaojun Chen, Heinrich Mächler, Jan Stefan Kirschke, Ezequiel de la Rosa, Patrick Ferdinand Christ, Hongwei Bran Li, David G. Ellis, Michele R. Aizenberg, Sergios Gatidis, Thomas Küstner, Nadya Shusharina, Nicholas Heller, Vincent Andrearczyk, Adrien Depeursinge, Mathieu Hatt, Anjany Sekuboyina, Maximilian T. Löffler, Hans Liebl, Reuben Dorent, Tom Vercauteren, Jonathan Shapey, Aaron Kujawa, Stefan Cornelissen, Patrick Langenhuizen, Achraf Ben-Hamadou, Ahmed Rekik, Sergi Pujades, Edmond Boyer, Federico Bolelli, Costantino Grana, Luca Lumetti, Hamidreza Salehi, Jun Ma, Yao Zhang, Ramtin Gharleghi, Susann Beier, Arcot Sowmya, Eduardo A. Garza-Villarreal, Thania Balducci, Diego Angeles-Valdez, Roberto Souza, Leticia Rittner, Richard Frayne, Yuanfeng Ji, Vincenzo Ferrari, Soumick Chatterjee, Florian Dubost, Stefanie Schreiber, Hendrik Mattern, Oliver Speck, Daniel Haehn, Christoph John, Andreas Nürnberger, João Pedrosa, Carlos Ferreira, Guilherme Aresta, António Cunha, Aurélio Campilho, Yannick Suter, Jose Garcia, Alain Lalande, Vicky Vandenbossche, Aline Van Oevelen, Kate Duquesne, Hamza Mekhzoum, Jef Vandemeulebroucke, Emmanuel Audenaert, Claudia Krebs, Timo van Leeuwen, Evie Vereecke, Hauke Heidemeyer, Rainer Röhrig, Frank Hölzle, Vahid Badeli, Kathrin Krieger, Matthias Gunzer, Jianxu Chen, Timo van Meegdenburg, Amin Dada, Miriam Balzer, Jana Fragemann, Frederic Jonske, Moritz Rempe, Stanislav Malorodov, Fin H. Bahnsen, Constantin Seibold, Alexander Jaus, Zdravko Marinov, Paul F. Jaeger, Rainer Stiefelhagen, Ana Sofia Santos, Mariana Lindo, André Ferreira, Victor Alves, Michael Kamp, Amr Abourayya, Felix Nensa, Fabian Hörst, Alexander Brehmer, Lukas Heine, Yannik Hanusrichter, Martin Weßling, Marcel Dudda, Lars E. Podleska, Matthias A. Fink, Julius Keyl, Konstantinos Tserpes, Moon-Sung Kim, Shireen Elhabian, Hans Lamecker, Dženan Zukić, Beatriz Paniagua, Christian Wachinger, Martin Urschler, Luc Duong, Jakob Wasserthal, Peter F. Hoyer, Oliver Basu, Thomas Maal, Max J. H. Witjes, Gregor Schiele, Ti-chiun Chang, Seyed-Ahmad Ahmadi, Ping Luo, Bjoern Menze, Mauricio Reyes, Thomas M. Deserno, Christos Davatzikos, Behrus Puladi, Pascal Fua, Alan L. Yuille, Jens Kleesiek, Jan Egger

**Affiliations:** Institute for Artificial Intelligence in Medicine (IKIM), University Hospital Essen (AöR), Essen, Germany; Institute of Computer Graphics and Vision (ICG), Graz University of Technology, Graz, Austria; and Computer Algorithms for Medicine Laboratory (Cafe), Graz, Austria; Department of Computer Science, Johns Hopkins University, Baltimore, MD, USA; Computer Vision Laboratory, Swiss Federal Institute of Technology Lausanne (EPFL), Lausanne, Switzerland; Institute of Computer Graphics and Vision (ICG), Graz University of Technology, Graz, Austria; and Computer Algorithms for Medicine Laboratory (Cafe), Graz, Austria; Institute of Computer Graphics and Vision (ICG), Graz University of Technology, Graz, Austria; Computer Algorithms for Medicine Laboratory (Cafe), Graz, Austria; and Department of Oral and Maxillofacial Surgery, University Hospital RWTH Aachen, Aachen, Germany; Institute for Artificial Intelligence in Medicine (IKIM), University Hospital Essen (AöR), Essen, Germany; Institute of Computer Graphics and Vision (ICG), Graz University of Technology, Graz, Austria; Computer Algorithms for Medicine Laboratory (Cafe), Graz, Austria; and Center for Virtual and Extended Reality in Medicine (ZvRM), University Hospital Essen, University Medicine Essen, Essen, Germany; Department of Computer Science, Johns Hopkins University, Baltimore, MD, USA; Department of Computer Science, Johns Hopkins University, Baltimore, MD, USA; Department of Radiology, Renji Hospital, School of Medicine, Shanghai Jiao Tong University, Shanghai, China; Department of Computer Science, Johns Hopkins University, Baltimore, MD, USA; Department of Measurement and Electronics, AGH University of Science and Technology, Krakow, Poland; and Information Systems Institute, University of Applied Sciences Western Switzerland (HES-SO Valais), Sierre, Switzerland; Center for Medical Image Analysis & Navigation (CIAN), Department of Biomedical Engineering, University of Basel, Allschwil, Switzerland; Department of Computer Science and Engineering, University at Buffalo, SUNY, NY, 14260, USA; Institute of Computer Graphics and Vision (ICG), Graz University of Technology, Graz, Austria; Computer Algorithms for Medicine Laboratory (Cafe), Graz, Austria; and Research Center for Connected Healthcare Big Data, ZhejiangLab, Hangzhou, Zhejiang, China; Institute for Artificial Intelligence in Medicine (IKIM), University Hospital Essen (AöR), Essen, Germany; Institute for Artificial Intelligence in Medicine (IKIM), University Hospital Essen (AöR), Essen, Germany; Institute of Computer Graphics and Vision (ICG), Graz University of Technology, Graz, Austria; and Computer Algorithms for Medicine Laboratory (Cafe), Graz, Austria; Institute of Mechanics, Graz University of Technology, Graz, Austria; Department of Diagnostic and Interventional Radiology, University Hospital Giessen, Justus-Liebig-University Giessen, Giessen, Germany; Department of Mechanical Engineering, Mehran University of Engineering and Technology, Jamshoro, Sindh, Pakistan; and Institute of Medical Robotics, Shanghai Jiao Tong University, Shanghai, People’s Republic of China; Canadian Surgical Technologies & Advanced Robotics (CSTAR), University Hospital, London, Canada; Canadian Surgical Technologies & Advanced Robotics (CSTAR), University Hospital, London, Canada; Canadian Surgical Technologies & Advanced Robotics (CSTAR), University Hospital, London, Canada; Canadian Surgical Technologies & Advanced Robotics (CSTAR), University Hospital, London, Canada; State Key Laboratory of Mechanical System and Vibration, School of Mechanical Engineering, Institute of Biomedical Manufacturing and Life Quality Engineering, Shanghai Jiao Tong University, Shanghai, People’s Republic of China; and Institute of Medical Robotics, Shanghai Jiao Tong University, Shanghai, People’s Republic of China; Department of Cardiac Surgery, Medical University Graz, Graz, Austria; Geschäftsführender Oberarzt Abteilung für Interventionelle und Diagnostische Neuroradiologie, Universitätsklinikum der Technischen Universität München, München, Germany; icometrix, Leuven, Belgium; and Department of Informatics, Technical University of Munich, Garching bei München, Germany; Department of Quantitative Biomedicine, University of Zurich, Zurich, Switzerland; Department of Quantitative Biomedicine, University of Zurich, Zurich, Switzerland; Department of Neurosurgery, University of Nebraska Medical Center, Omaha, NE, USA; Department of Neurosurgery, University of Nebraska Medical Center, Omaha, NE, USA; University Hospital of Tuebingen Diagnostic and Interventional Radiology Medical Image and Data Analysis (MIDAS.lab), Tuebingen, Germany; University Hospital of Tuebingen Diagnostic and Interventional Radiology Medical Image and Data Analysis (MIDAS.lab), Tuebingen, Germany; Division of Radiation Biophysics, Department of Radiation Oncology, Massachusetts General Hospital and Harvard Medical School, Boston, MA, USA; University of Minnesota, Minneapolis, MN, USA; Institute of Informatics, HES-SO Valais-Wallis University of Applied Sciences and Arts Western Switzerland, Sierre, Switzerland; Institute of Informatics, HES-SO Valais-Wallis University of Applied Sciences and Arts Western Switzerland, Sierre, Switzerland; and Department of Nuclear Medicine and Molecular Imaging, Lausanne University Hospital (CHUV), Lausanne, Switzerland; LaTIM, INSERM UMR 1101, Univ Brest, Brest, France; Department of Informatics, Technical University of Munich, Garching bei München, Germany; Department of Neuroradiology, Klinikum Rechts der Isar, Munich, Germany; Department of Neuroradiology, Klinikum Rechts der Isar, Munich, Germany; King’s College London, Strand, London, UK; and Department of Neurosurgery, Brigham and Women’s Hospital, Harvard Medical School, Boston, MA, USA; King’s College London, Strand, London, UK; King’s College London, Strand, London, UK; King’s College London, Strand, London, UK; Elisabeth-TweeSteden Hospital, Tilburg, Netherlands; and Video Coding & Architectures Research Group, Department of Electrical Engineering, Eindhoven University of Technology, Eindhoven, Netherlands; Elisabeth-TweeSteden Hospital, Tilburg, Netherlands; and Video Coding & Architectures Research Group, Department of Electrical Engineering, Eindhoven University of Technology, Eindhoven, Netherlands; Centre de Recherche en Numérique de Sfax, Laboratory of Signals, Systems, Artificial Intelligence and Networks, Sfax, Tunisia; and Udini, Aix-en-Provence, France; Centre de Recherche en Numérique de Sfax, Laboratory of Signals, Systems, Artificial Intelligence and Networks, Sfax, Tunisia; and Udini, Aix-en-Provence, France; Inria, Université Grenoble Alpes, CNRS, Grenoble, France; Inria, Université Grenoble Alpes, CNRS, Grenoble, France; “Enzo Ferrari” Department of Engineering, University of Modena and Reggio Emilia, Modena, Italy; “Enzo Ferrari” Department of Engineering, University of Modena and Reggio Emilia, Modena, Italy; “Enzo Ferrari” Department of Engineering, University of Modena and Reggio Emilia, Modena, Italy; Department of Artificial Intelligence in Medical Sciences, Faculty of Advanced Technologies in Medicine, Iran University of Medical Sciences, Tehran, Iran; Department of Laboratory Medicine and Pathobiology, University of Toronto, Toronto, ON, Canada; Peter Munk Cardiac Centre, University Health Network, Toronto, ON, Canada; and Vector Institute, Toronto, ON, Canada; Shanghai AI Laboratory, Shanghai, People’s Republic of China; School of Mechanical and Manufacturing Engineering, UNSW, Sydney, NSW, Australia; School of Mechanical and Manufacturing Engineering, UNSW, Sydney, NSW, Australia; School of Computer Science and Engineering, UNSW, Sydney, NSW, Australia; Institute of Neurobiology, Universidad Nacional Autónoma de México Campus Juriquilla, Querétaro, Mexico; Institute of Neurobiology, Universidad Nacional Autónoma de México Campus Juriquilla, Querétaro, Mexico; Institute of Neurobiology, Universidad Nacional Autónoma de México Campus Juriquilla, Querétaro, Mexico; and Department of Biomedical Sciences of Cells and Systems, Cognitive Neuroscience Center, University Medical Center Groningen, University of Groningen, Groningen, Netherlands; Advanced Imaging and Artificial Intelligence Lab, Electrical and Software Engineering Department, The Hotchkiss Brain Institute, University of Calgary, Calgary, Canada; Medical Image Computing Lab, School of Electrical and Computer Engineering (FEEC), University of Campinas, Campinas, Brazil; Radiology and Clinical Neurosciences Departments, The Hotchkiss Brain Institute, University of Calgary, Calgary, Canada; and Seaman Family MR Research Centre, Foothills Medical Center, Calgary, Canada; University of Hongkong, Pok Fu Lam, Hong Kong, People’s Republic of China; Dipartimento di Ingegneria dell’Informazione, University of Pisa, Pisa, Italy; and EndoCAS Center, Department of Translational Research and of New Surgical and Medical Technologies, University of Pisa, Pisa, Italy; Genomics Research Centre, Human Technopole, Milan, Italy; and Data and Knowledge Engineering Group, Faculty of Computer Science, Otto von Guericke University Magdeburg, Magdeburg, Germany; Stanford University, Stanford, CA, USA; Department of Neurology, Medical Faculty, University Hospital of Magdeburg, Magdeburg, Germany; German Centre for Neurodegenerative Disease, Magdeburg, Germany; and Centre for Behavioural Brain Sciences, Magdeburg, Germany; Department of Biomedical Magnetic Resonance, Otto von Guericke University Magdeburg, Magdeburg, Germany; German Centre for Neurodegenerative Disease, Magdeburg, Germany; and Centre for Behavioural Brain Sciences, Magdeburg, Germany; Department of Biomedical Magnetic Resonance, Otto von Guericke University Magdeburg, Magdeburg, Germany; German Centre for Neurodegenerative Disease, Magdeburg, Germany; and Centre for Behavioural Brain Sciences, Magdeburg, Germany; University of Massachusetts Boston, Boston, MA, USA; Ecubed Solutions, Bensheim, Germany; Data and Knowledge Engineering Group, Faculty of Computer Science, Otto von Guericke University Magdeburg, Magdeburg, Germany; and Centre for Behavioural Brain Sciences, Magdeburg, Germany; Institute for Systems and Computer Engineering, Technology and Science (INESC TEC), Porto, Portugal; and Faculty of Engineering, University of Porto (FEUP), Porto, Portugal; Institute for Systems and Computer Engineering, Technology and Science (INESC TEC), Porto, Portugal; and Faculty of Engineering, University of Porto (FEUP), Porto, Portugal; Christian Doppler Lab for Artificial Intelligence in Retina, Department of Ophthalmology and Optometry, Medical University of Vienna, Vienna, Austria; Institute for Systems and Computer Engineering, Technology and Science (INESC TEC), Porto, Portugal; and Universidade of Trás-os-Montes and Alto Douro (UTAD), Vila Real, Portugal; Institute for Systems and Computer Engineering, Technology and Science (INESC TEC), Porto, Portugal; and Faculty of Engineering, University of Porto (FEUP), Porto, Portugal; ARTORG Center for Biomedical Engineering Research, University of Bern, Bern, Switzerland; Center for Biomedical Image Computing and Analytics (CBICA), Perelman School of Medicine, University of Pennsylvania, Philadelphia, USA; ICMUB Laboratory, Faculty of Medicine, CNRS UMR 6302, University of Burgundy, Dijon, France; and Medical Imaging Department, University Hospital of Dijon, Dijon, France; Department of Human Structure and Repair, Ghent University, Ghent, Belgium; Department of Human Structure and Repair, Ghent University, Ghent, Belgium; Department of Human Structure and Repair, Ghent University, Ghent, Belgium; Department of Electronics and Informatics (ETRO), Vrije Universiteit Brussel, Brussels, Belgium; Department of Electronics and Informatics (ETRO), Vrije Universiteit Brussel, Brussels, Belgium; Department of Human Structure and Repair, Ghent University, Ghent, Belgium; Department of Cellular and Physiological Sciences, Life Sciences Centre, University of British Columbia, Vancouver, BC, Canada; Department of Development & Regeneration, KU Leuven Campus Kulak, Kortrijk, Belgium; Department of Development & Regeneration, KU Leuven Campus Kulak, Kortrijk, Belgium; Institute of Medical Informatics, University Hospital RWTH Aachen, Aachen, Germany; Institute of Medical Informatics, University Hospital RWTH Aachen, Aachen, Germany; Department of Oral and Maxillofacial Surgery, University Hospital RWTH Aachen, Aachen, Germany; Institute of Fundamentals and Theory in Electrical Engineering, Graz University of Technology, Graz, Austria; Leibniz-Institut für Analytische Wissenschaften-ISAS-e.V., Dortmund, Germany; Leibniz-Institut für Analytische Wissenschaften-ISAS-e.V., Dortmund, Germany; and Institute for Experimental Immunology and Imaging, University Hospital, University Duisburg-Essen, Essen, Germany; Leibniz-Institut für Analytische Wissenschaften-ISAS-e.V., Dortmund, Germany; Institute for Artificial Intelligence in Medicine (IKIM), University Hospital Essen (AöR), Essen, Germany; and Faculty of Statistics, Technical University Dortmund, Dortmund, Germany; Institute for Artificial Intelligence in Medicine (IKIM), University Hospital Essen (AöR), Essen, Germany; Institute for Artificial Intelligence in Medicine (IKIM), University Hospital Essen (AöR), Essen, Germany; Institute for Artificial Intelligence in Medicine (IKIM), University Hospital Essen (AöR), Essen, Germany; Institute for Artificial Intelligence in Medicine (IKIM), University Hospital Essen (AöR), Essen, Germany; Institute for Artificial Intelligence in Medicine (IKIM), University Hospital Essen (AöR), Essen, Germany; Institute for Artificial Intelligence in Medicine (IKIM), University Hospital Essen (AöR), Essen, Germany; Institute for Artificial Intelligence in Medicine (IKIM), University Hospital Essen (AöR), Essen, Germany; Institute for Artificial Intelligence in Medicine (IKIM), University Hospital Essen (AöR), Essen, Germany; Computer Vision for Human-Computer Interaction Lab, Department of Informatics, Karlsruhe Institute of Technology, Karlsruhe, Germany; Computer Vision for Human-Computer Interaction Lab, Department of Informatics, Karlsruhe Institute of Technology, Karlsruhe, Germany; German Cancer Research Center (DKFZ) Heidelberg, Interactive Machine Learning Group, Heidelberg, Germany; and Helmholtz Imaging, DKFZ Heidelberg, Heidelberg, Germany; Computer Vision for Human-Computer Interaction Lab, Department of Informatics, Karlsruhe Institute of Technology, Karlsruhe, Germany; Institute for Artificial Intelligence in Medicine (IKIM), University Hospital Essen (AöR), Essen, Germany; and Center Algoritmi, LASI, University of Minho, Braga, Portugal; Institute for Artificial Intelligence in Medicine (IKIM), University Hospital Essen (AöR), Essen, Germany; and Center Algoritmi, LASI, University of Minho, Braga, Portugal; Institute for Artificial Intelligence in Medicine (IKIM), University Hospital Essen (AöR), Essen, Germany; and Center Algoritmi, LASI, University of Minho, Braga, Portugal; Center Algoritmi, LASI, University of Minho, Braga, Portugal; Institute for Artificial Intelligence in Medicine (IKIM), University Hospital Essen (AöR), Essen, Germany; Cancer Research Center Cologne Essen (CCCE), University Medicine Essen (AöR), Essen, Germany; Institute for Neuroinformatics, Ruhr University Bochum, Bochum, Germany; and Department of Data Science & AI, Monash University, Clayton, VIC, Australia; Institute for Artificial Intelligence in Medicine (IKIM), University Hospital Essen (AöR), Essen, Germany; and Institute for Neuroinformatics, Ruhr University Bochum, Bochum, Germany; Institute for Artificial Intelligence in Medicine (IKIM), University Hospital Essen (AöR), Essen, Germany; and Institute of Diagnostic and Interventional Radiology and Neuroradiology, University Hospital Essen (AöR), Essen, Germany; Institute for Artificial Intelligence in Medicine (IKIM), University Hospital Essen (AöR), Essen, Germany; and Cancer Research Center Cologne Essen (CCCE), University Medicine Essen (AöR), Essen, Germany; Institute for Artificial Intelligence in Medicine (IKIM), University Hospital Essen (AöR), Essen, Germany; Institute for Artificial Intelligence in Medicine (IKIM), University Hospital Essen (AöR), Essen, Germany; and Cancer Research Center Cologne Essen (CCCE), University Medicine Essen (AöR), Essen, Germany; Department of Tumour Orthopaedics and Revision Arthroplasty, Orthopaedic Hospital Volmarstein, Wetter, Germany; and Center for Musculoskeletal Surgery, University Hospital of Essen, Essen, Germany; Department of Tumour Orthopaedics and Revision Arthroplasty, Orthopaedic Hospital Volmarstein, Wetter, Germany; and Center for Musculoskeletal Surgery, University Hospital of Essen, Essen, Germany; Department of Trauma, Hand and Reconstructive Surgery, University Hospital Essen, Essen, Germany; and Department of Orthopaedics and Trauma Surgery, BG-Klinikum Duisburg, University of Duisburg-Essen, Essen, Germany; Department of Tumor Orthopedics and Sarcoma Surgery, University Hospital Essen (AöR), Essen, Germany; Clinic for Diagnostic and Interventional Radiology, University Hospital Heidelberg, Heidelberg, Germany; Institute for Artificial Intelligence in Medicine (IKIM), University Hospital Essen (AöR), Essen, Germany; Department of Informatics and Telematics, Harokopio University of Athens, Tavros, Greece; Institute for Artificial Intelligence in Medicine (IKIM), University Hospital Essen (AöR), Essen, Germany; Institute of Diagnostic and Interventional Radiology and Neuroradiology, University Hospital Essen (AöR), Essen, Germany; and Cancer Research Center Cologne Essen (CCCE), University Medicine Essen (AöR), Essen, Germany; Scientific Computing and Imaging Institute, University of Utah, Salt Lake City, USA; Stryker Berlin GmbH, Germany; Medical Computing, Kitware Inc., Carrboro, NC, USA; Medical Computing, Kitware Inc., Carrboro, NC, USA; Lab for Artificial Intelligence in Medical Imaging, Department of Radiology, Technical University Munich, Munich, Germany; Institute for Medical Informatics, Statistics and Documentation, Medical University Graz, Graz, Austria; Department of Software and IT Engineering, Ecole de Technologie Superieure, Montreal, Quebec, Canada; Clinic of Radiology & Nuclear Medicine, University Hospital Basel, Basel, Switzerland; Pediatric Clinic II, University Children’s Hospital Essen, University Duisburg-Essen, Essen, Germany; Pediatric Clinic III, University Children’s Hospital Essen, University Duisburg-Essen, Essen, Germany; and Center for Virtual and Extended Reality in Medicine (ZvRM), University Hospital Essen, University Medicine Essen, Essen, Germany; Radboudumc 3D-Lab, Department of Oral and Maxillofacial Surgery, Radboud University Nijmegen Medical Centre, Nijmegen, The Netherlands; 3D Lab, Department of Oral and Maxillofacial Surgery, University Medical Center Groningen, Groningen, the Netherlands; Intelligent Embedded Systems Lab, University of Duisburg-Essen, Bismarckstraße 90, 47057 Duisburg, Germany; MRL, Merck & Co., Inc., Rahway, NJ 07065, USA; NVIDIA GmbH, Bavaria Towers – Blue Tower, Munich, Germany; University of Hongkong, Pok Fu Lam, Hong Kong, People’s Republic of China; Department of Quantitative Biomedicine, University of Zurich, Zurich, Switzerland; ARTORG Center for Biomedical Engineering Research, University of Bern, Bern, Switzerland; and Department of Radiation Oncology, University Hospital Bern, University of Bern, Bern, Switzerland; Peter L. Reichertz Institute for Medical Informatics of TU Braunschweig and Hannover Medical School, Braunschweig, Germany; Center for Biomedical Image Computing and Analytics, Penn Neurodegeneration Genomics Center, University of Pennsylvania, Philadelphia, PA, USA; and Center for AI and Data Science for Integrated Diagnostics, University of Pennsylvania, Philadelphia, PA, USA; Department of Oral and Maxillofacial Surgery, University Hospital RWTH Aachen, Aachen, Germany; and Institute of Medical Informatics, University Hospital RWTH Aachen, Aachen, Germany; Computer Vision Laboratory, Swiss Federal Institute of Technology Lausanne (EPFL), Lausanne, Switzerland; Department of Computer Science, Johns Hopkins University, Baltimore, MD, USA; Institute for Artificial Intelligence in Medicine (IKIM), University Hospital Essen (AöR), Essen, Germany; German Cancer Consortium (DKTK), Partner Site Essen, Essen, Germany; Department of Physics, TU Dortmund University, Dortmund, Germany; and Cancer Research Center Cologne Essen (CCCE), University Medicine Essen (AöR), Essen, Germany; Institute for Artificial Intelligence in Medicine (IKIM), University Hospital Essen (AöR), Girardetstraße 2, 45131 Essen, Germany; Institute of Computer Graphics and Vision (ICG), Graz University of Technology, Inffeldgasse 16c, 8010 Graz, Austria; Computer Algorithms for Medicine Laboratory (Cafe), Graz, Austria; Cancer Research Center Cologne Essen (CCCE), University Medicine Essen (AöR), Hufelandstraße 55, 45147 Essen, Germany; and Center for Virtual and Extended Reality in Medicine (ZvRM), University Hospital Essen, University Medicine Essen, Hufelandstraße 55, 45147 Essen, Germany

**Keywords:** 3D medical shapes, benchmark, anatomy education, shapeomics, augmented reality, virtual reality

## Abstract

**Objectives:**

The shape is commonly used to describe the objects. State-of-the-art algorithms in medical imaging are predominantly diverging from computer vision, where voxel grids, meshes, point clouds, and implicit surface models are used. This is seen from the growing popularity of ShapeNet (51,300 models) and Princeton ModelNet (127,915 models). However, a large collection of anatomical shapes (e.g., bones, organs, vessels) and 3D models of surgical instruments is missing.

**Methods:**

We present MedShapeNet to translate data-driven vision algorithms to medical applications and to adapt state-of-the-art vision algorithms to medical problems. As a unique feature, we directly model the majority of shapes on the imaging data of real patients. We present use cases in classifying brain tumors, skull reconstructions, multi-class anatomy completion, education, and 3D printing.

**Results:**

By now, MedShapeNet includes 23 datasets with more than 100,000 shapes that are paired with annotations (ground truth). Our data is freely accessible via a web interface and a Python application programming interface and can be used for discriminative, reconstructive, and variational benchmarks as well as various applications in virtual, augmented, or mixed reality, and 3D printing.

**Conclusions:**

MedShapeNet contains medical shapes from anatomy and surgical instruments and will continue to collect data for benchmarks and applications. The project page is: https://medshapenet.ikim.nrw/.

## Introduction

The success of deep learning in many fields of applications, including vision [1], language [2] and speech [3], is mainly due to the availability of large, high-quality datasets [4–6], such as *ImageNet* [7], *CIFAR* [8], *Penn Treebank* [9], *WikiText* [10] and *LibriSpeech* [11]. In 3D computer vision, *Princeton ModelNet* [12], *ShapeNet* [13], etc., are the *de facto* benchmarks for numerous fundamental vision problems, including 3D shape classification and retrieval [14], shape completion [15], shape reconstruction and segmentation [16]. Shape describes the geometries of 3D objects and is one of the most basic concepts in computer vision. Common 3D shape representations include point clouds, voxel occupancy grids, meshes, and implicit surface models (signed distance functions), which follow different data structures, cater for different algorithms, and are convertible to each other [17]. These shape representations diverge from gray-scale medical imaging data routinely used in clinical diagnosis and treatment procedures, such as computed tomography (CT), magnetic resonance imaging (MRI), positron emission tomography (PET), ultra sound (US), and X-ray.

The concept of shape in medical imaging is not novel. For example, statistical shape modeling (SSM) has been a longstanding method for medical image segmentation [18] and 3D anatomy modeling [19]. The use of shape priors and constraints can also benefit medical image segmentation and reconstruction tasks [20]. Furthermore, the prominent *Medical Image Computing and Computer Assisted Intervention (MICCAI)* society has established a special interest group in *Shape in Medical Imaging* (*ShapeMI*). This group is dedicated to exploring the applications of both traditional and contemporary (e.g., learning-based) shape analysis methods in medical imaging. Table 1 presents a partial list of professional organizations and events that are committed to this objective.

Nevertheless, state-of-the-art (SOTA) algorithms connot be directly applied to medical problems, since the vision methods were developed on general 3D shapes from *ShapeNet* and not on volumetric, gray-scale medical data. Therefore, the community needs a large, high-quality shape database for medical imaging that represents a variety of 3D medical shapes, i.e., voxel occupancy grid (VOR), mesh and point representations of human anatomies [21]. The inclusion of diverse anatomical shapes can aid in the development and evaluation of data-driven, shape-based methods for both vision and medical problems.

Computer vision methods, such as facial modeling [22] and internal anatomy inference [23] involve anatomical shapes, and medical problems can be solved using shape-based methods. Cranial implant design [24–28] is a typical example of a clinical problem that is commonly solved using well-established shape completion methods [29]. Such a shape completion concept can also be straightforwardly extended to other anatomical structures or even the whole body [30]. Therefore, there is a need for both normal and pathological anatomies to solve shape-based problems that are conventionally addressed using gray-scale medical images, e.g., extacting biomarkers [31].

In this paper, we present *MedShapeNet*, (1) a unique dataset for medical imaging shapes that serve complementary to existing shape benchmarks in computer vision, (2) a gap-bridger between the medical imaging and computer vision communities, and (3) a publicly available, continuous extending resource for benchmarking, education, extended reality (XR) applications [32], and the investigation of anatomical shape variations.

**Figure 1:**
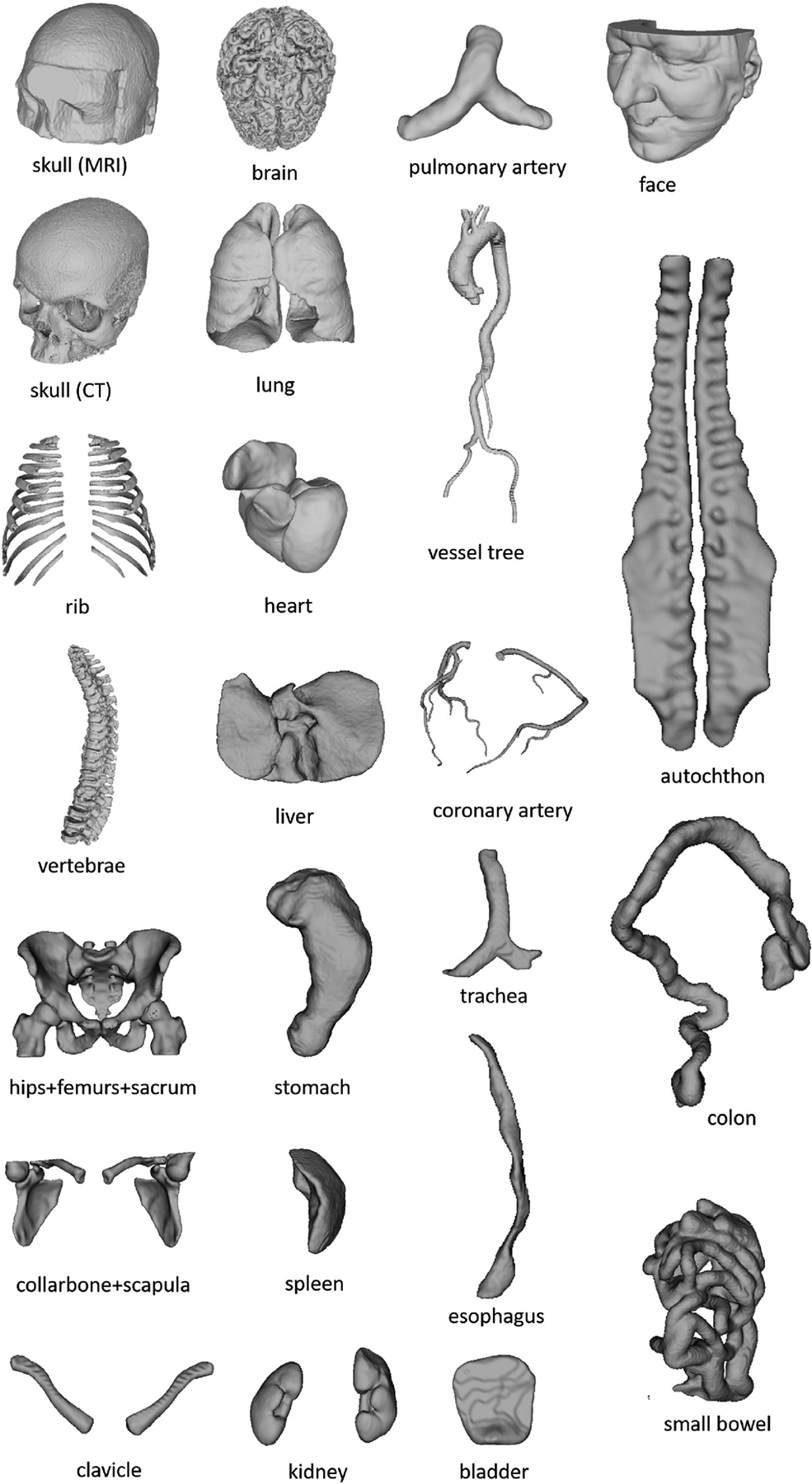
Example shapes in *MedShapeNet*, including various bones (e.g., skulls, ribs and vertebrae), organs (e.g., brain, lung, heart, liver), vessels (e.g., aortic vessel tree and pulmonary artery) and muscles.

While existing datasets, such as *ShapeNet* are comprised of 3D computer-aided design (CAD) models of real-world objects (e.g., *plane*, *car*, *chair*, *desk*), *MedShapeNet* provides 3D shapes extracted from the imaging data of real patients including healthy as well as pathological subjects (Figure 1).

## Shape and voxel features

**Figure 2:**
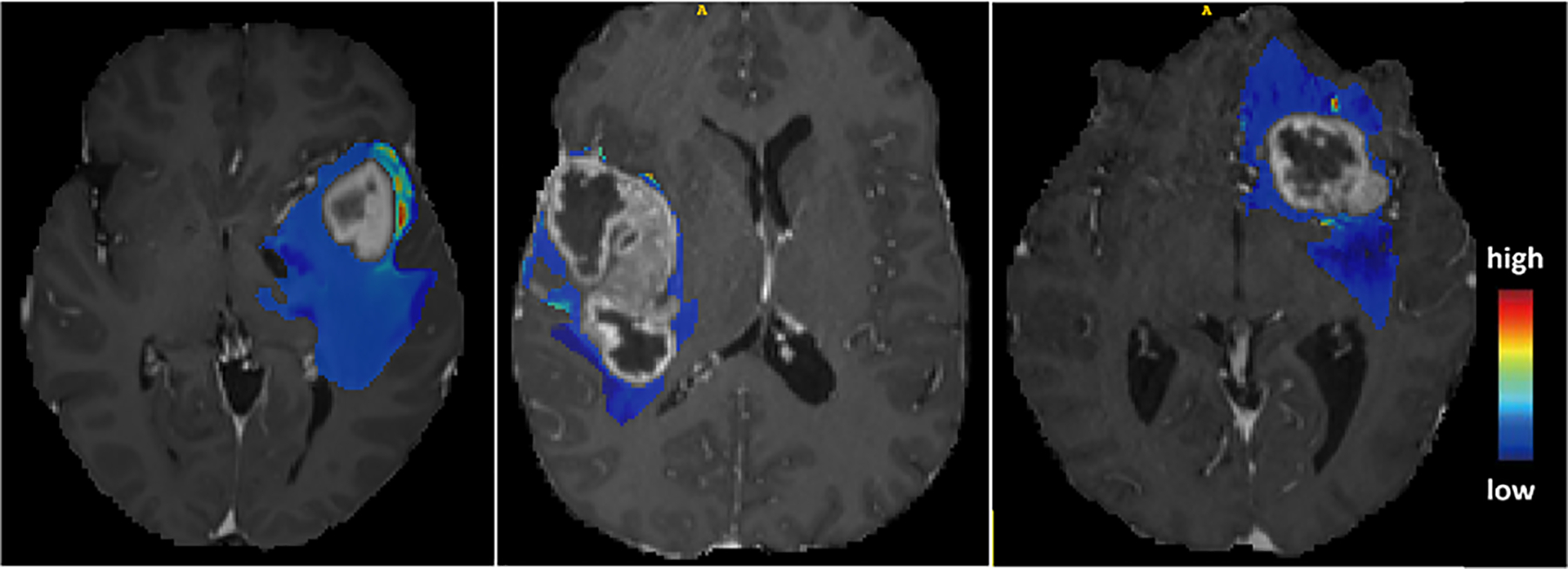
The *predictive maps* overlaid onto patients’ MRI scans. The *predictive maps* are color-coded to indicate high or low probability of tumor infiltration.

**Figure 3:**
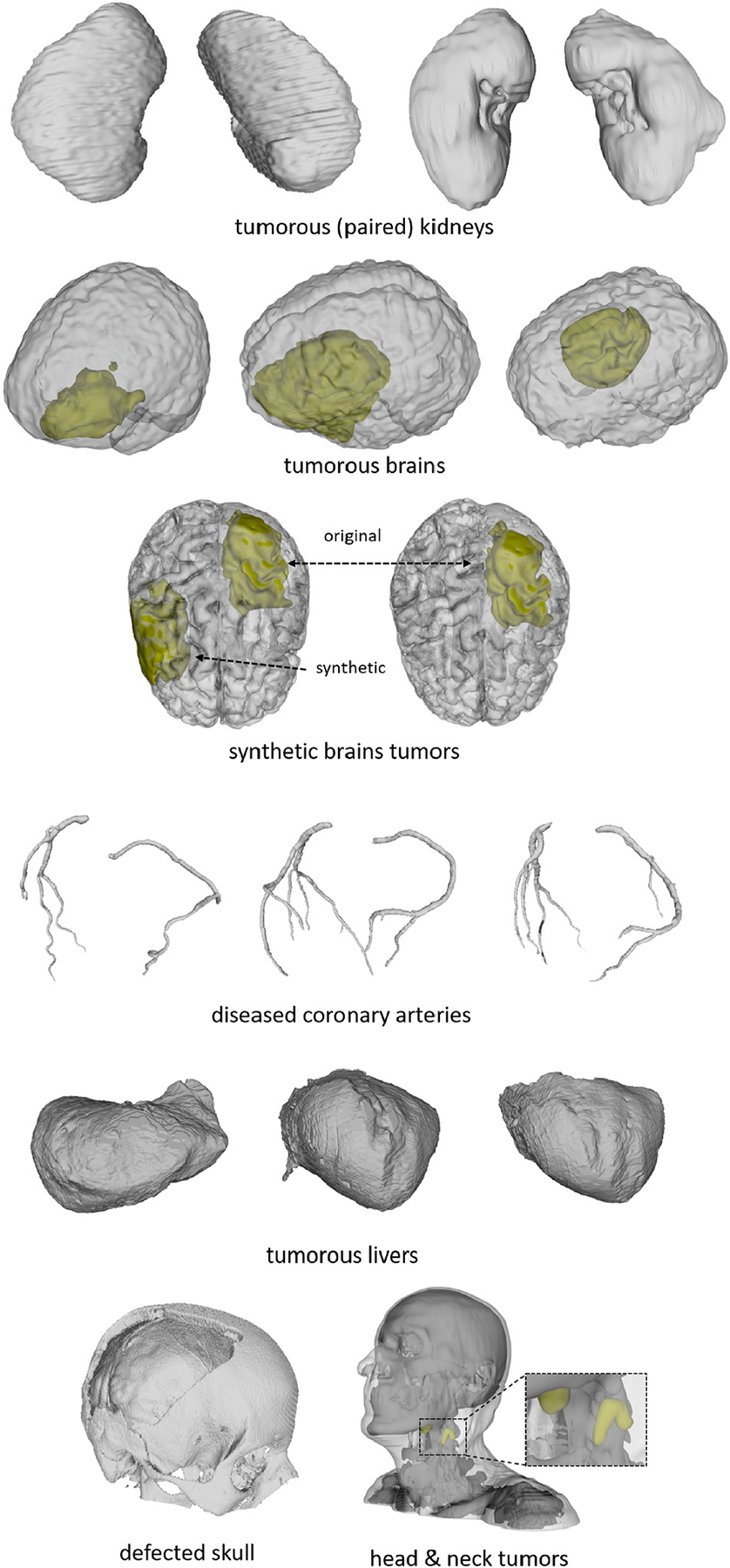
Example pathological shapes in *MedShapeNet*, including tumorous kidney (paired), brain (with real and synthetic tumors), liver and head & neck, as well as diseased coronary arteries. For illustration purpose, the opacity of some shapes is reduced to reveal the underlying tumors. We can study the effects of tumors on the morphological changes of an anatomy (e.g., brain) using such pathological data.

Shapes describe objects’ geometries, provide a foundation for computer vision, and serve as a computationally efficient way to represent images despite not capturing voxel features. In medicine, numerous diseases alter the morphological attributes of the affected anatomical structures (Figure 2). For instance, neoplastic formations, such as tumors, significant alter the morphologies of organs like the brain and the liver (Figure 3); neurological disorders, including Alzheimer’s disease (AD) [33], Parkinson’s disease (PD) [34] and substance use disorders, for instance, alcohol use disorder (AUD) and cocaine use disorder (CUD), can also cause morphological changes of brain substructures, such as the cerebral ventricles and the subcortical structures. These morphologic alterations allow disease detection and classification either manually, by medical professionals or automatically, through the application of specialized (e.g., shape analysis) machine learning algorithms.

Hence, *MedShapeNet* highlights the significance of shape features, including jaggedness, volume, elongation, etc., over voxel features, such as intensities, for disease characterization, current medical image analysis tasks are still dominated by voxel-based methods. For instance, the so-called voxel-wise spatial *predictive maps*, as demonstrated by Akbari et al. [35], can pinpoint areas of early recurrence and infiltration of glioblastoma. These maps can be effectively used for targeted radiotherapy [36] (Figure 2), as regions with high probability are associated with a greater risk of tumor recurrence after resection. A naturally arising question is whether such *predictive maps* can be derived from the tumors’ geometries. *MedShapeNet* provides a platform to investigate the question and more:

- What diseases can be comprehensively characterized by the shape features of the affected anatomical structures, and what diseases are solely reflected on voxel features?
- How can one obtain discriminative shape features for disease detection using a machine learning model, either by handcrafting or learning them automatically using a deep network?
- How to effectively combine shape and voxel features when shape features alone are insufficient for disease detection?
- Do changes in voxel and shape features correlate statistically, and if so, how can this correlation be quantified?
- Which of the current voxel-based mainstream approaches can be substituted with computationally more efficient shape-based methods for the analysis of medical data?

Transitioning from gray-scale imaging data to shape data and shape-based methods brings three primary benefits:

1. Shape manifolds are spatially sparse, which enables the use of more computationally efficient algorithms, such as sparse convolutions [37], point cloud [38] and mesh [39] neural networks;
2. Shape data contain less identifying information than gray-scale imaging data, reducing the vulnerability to privacy attack when they are publicly shared [40];
3. Training on shape data encourages a deep network to concentrate on learning discriminative geometric features instead of patients’ identities irrelevant to the task. This can help improve the robustness and trust-worthiness and prevent identity-driven bias of the learning system.

## Sources of shapes

**Figure 4:**
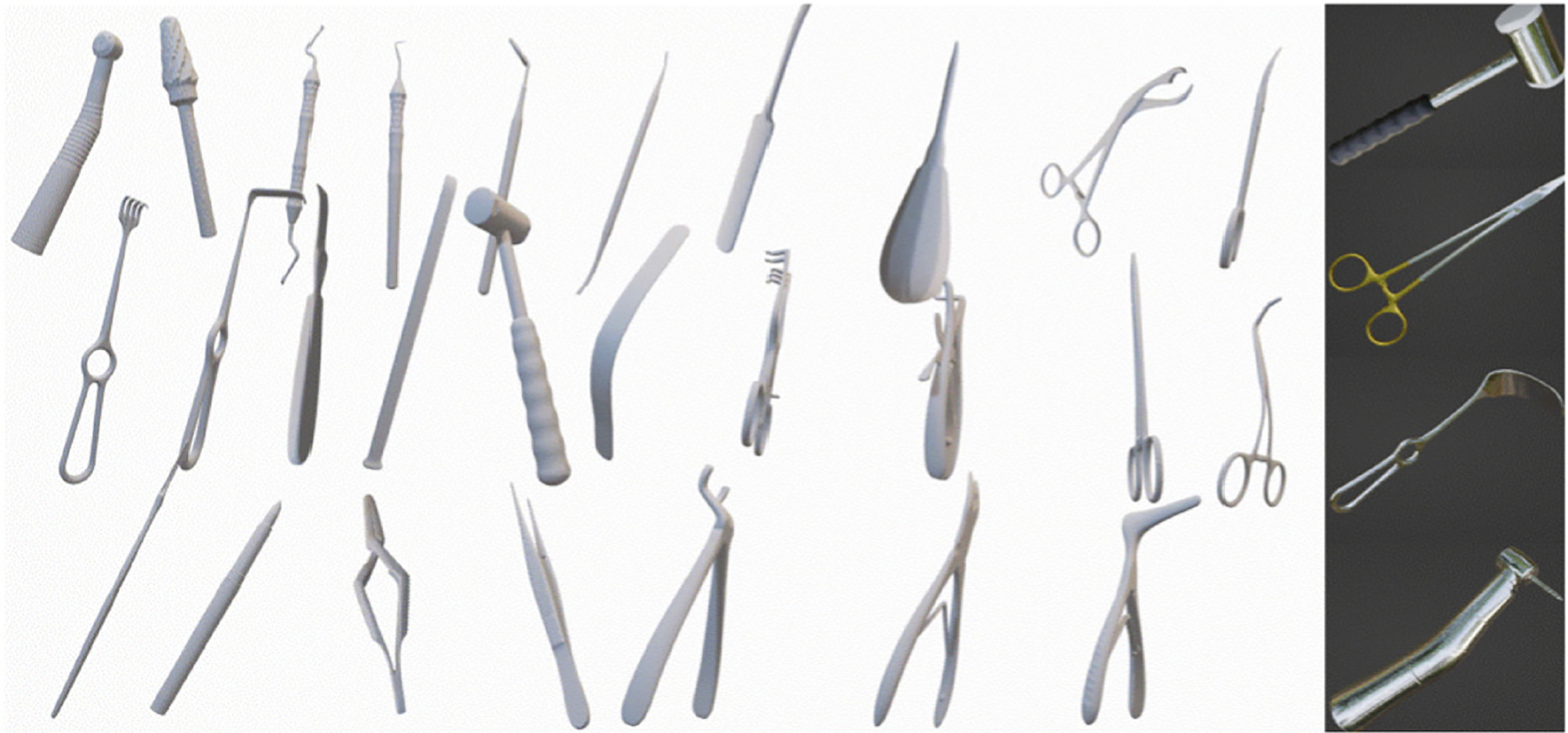
Illustration of 3D models of medical instruments used in oral and craniomaxillofacial surgeries. The 3D models are obtained using structured light 3D scanners (Artec Leo from Artec3D and AutoScan Inspec from shining 3D). Instrument models can be retrieved by the search query *instrument* via the *MedShapeNet* web interface. Image taken from https://xrlab.ikim.nrw/.

**Figure 5:**
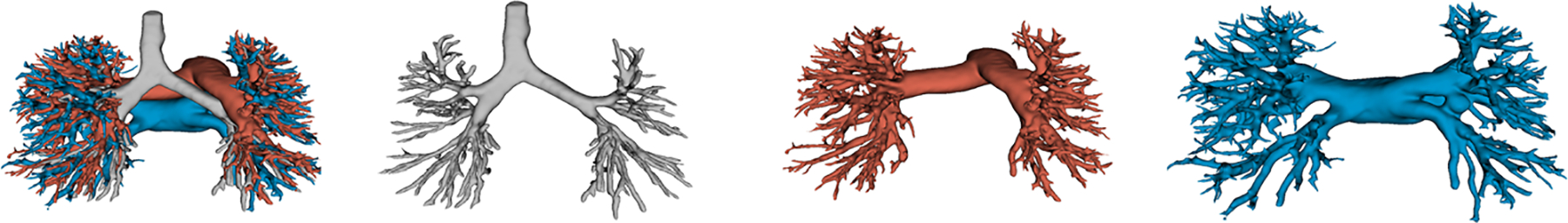
Illustration of a pulmonary tree comprising the airway, artery and vein – thin structures that are difficult to segment and reconstruct.

The shapes in *MedShapeNet* mostly originate from high-quality segmentation masks of anatomical structures, including different organs, bones, vessels, muscles, etc. (Figure 4). They are generated manually by domain experts, as those of the ground truth segmentations provided by medical image segmentation challenges [41], or semi-automatically, with the help of a segmentation network (e.g., *TotalSegmentator* [42], *autoPET whole-body segmentation* [43], *AbdomenAtlas* [44]). The majority of semi-automatic segmentations were also quality-checked by experts. Anatomical shapes with sophisticated geometric structures, such as the pulmonary trees (Figure 5), are also included in the *MedShapeNet* collection. In our terminology, we refer to binary voxel occupancy grids as segmentation masks, which we subsequently convert to meshes and point clouds using the *Marching Cubes* algorithm [45]. The majority of the source segmentation datasets are Creative Commons (CC) – licensed (Table 2), allowing us to adapt and redistribute the data. Furthermore, *MedShapeNet* includes both normal (Figure 1) and pathological shapes (Figure 3), delivered by the imaging data of healthy and diseased subjects, respectively. In addition, *MedShapeNet* provides 3D medical instrument models acquired using 3D handheld scanners [46] (Figure 4).

### AbdomenAtlas

The dataset provides masks of 25 anatomical structures and seven types of tumors, derived from 5,195 CTs of 26 hospitals across eight countries [44]. These anatomical structures include the spleen, right kidney, left kidney, gall bladder, esophagus, liver, stomach, aorta, postcava, portal and splenic veins, pancreas, right and left adrenal glands, duodenum, hepatic vessel, right and left lungs, colon, intestine, rectum, bladder, prostate, left and right femur heads, and celiac trunk. Shape quality is ensured through manual annotations by medical professionals supported by a semi-automatic active learning procedure. The pathology-confirmed tumors include kidney, liver, pancreatic, hepatic vessel, lung, colon, and kidney cysts. The dataset provides a total of 51.8 K tumor masks. Moreover, a novel modeling-based tumor synthesis method is used to generates small, synthetic (<20 mm) tumor shapes [92, 93].

### Pulmonary trees

The PulmonaryTree dataset [84] is a collection of pulmonary tree structures, amassed from 800 subjects across various medical centers in China [94]. It includes detailed 3D models of pulmonary airways, arteries, and veins, totaling 800 × 3=2,400 shapes. Each 3D model originates from CT scans with 512 × 512 voxels and 181–798 slices. The *Z*-spacing ranges from 0.5 to 1.5 mm. A collaborative annotation procedure ensures consistency provides a detailed and accurate representation of the pulmonary structures [95]. This procedure required approximately 3 h per case. The PulmonaryTree dataset introduces complex tree-like structures, a challenging aspect in medical image analysis (Figure 5). Specific technical challenges include maintaining the continuity of thin structures and addressing the uneven thickness of the main and branch structures.

### TotalSegmentator

The dataset from Wasserthal et al. [42] includes over 1,000 CT scans and the masks of 104 anatomical structures covering the whole body. The masks are generated automatically by a nnUNet [96]. The data have been used to improve diagnosis by correlating organ volumes with disease occurrences [97].

### Human connectome projects (HCP)

The *1,200 Subjects Data Release* from HCP includes 1,113 structural 3T head MRI scans of healthy young adults. From each scan, the *Cortical Surface Extraction* script provided by *BrainSuite*^1^ is used to extract the skull and brain masks.

### MUG500+

This dataset contains the binary masks and meshes of 500 healthy human skulls and 29 craniectomy skulls with surgical defects [81]. Thresholding delivered the masks from head CT scans.

### SkullBreak/SkullFix

The dataset includes the binary masks of healthy human skulls and the corresponding skulls with artificial defects. Similar to *MUG500*+ [81], thresholding head CTs from the *CQ500* dataset ^2^ yields the masks.

### Aortic vessel tree (AVT)

The dataset contains 56 computed tomography angiography (CTA) scans of healthy aortas and the masks of the aortic vessel trees [54], including the aorta, the aortic arch, the aortic branch, and the iliac arteries (Figure 1).

### Vertebrae segmentation (VerSe)

The *VerSe* challenge provides the masks of vertebrae from around 210 subjects [91]. In total, 2,745 vertebra shapes are generated.

### Automated segmentation of coronary arteries (ASOCA)

The *ASOCA* challenge provides the manual segmentations of 20 normal and 20 diseased coronary arteries [50].

### 3D teeth scan segmentation and labeling challenge (3DTeethSeg)

Automated teeth localization, segmentation, and labeling from intra-oral 3D scans significantly improve dental diagnostics, treatment planning, and population-based studies on oral health. Before initiating any orthodontic or restorative treatment, it is essential for a CAD system to accurately segment and label each instance of teeth. This eliminates the need of time-consuming manual adjustments by the dentist. The *3DTeethSeg* provides the upper and lower jaw scans of 900 subjects, and the manual segmentations of the teeth, obtained from clinical evaluators with more than 10 years of expertise [87, 88].

### Lung cancer patient management (LNDb) challenge

This dataset comprises lung nodule in low-dose CTs recorded for lung cancer screening [78, 79]. A total of 861 lung nodule masks correspond to 625 individual nodules segmented from 204 CTs. Five radiologists identified all pulmonary nodules with an in-plane dimension of 3 mm and higher.

### Evaluation of myocardial infarction from delayed-enhancement cardiac MRI (EMIDEC)

This *EMIDEC* challenge provides 150 delayed enhancement MRI (DE-MRI) images in short axis orientation of the left ventricles. Experts contoured the myocardium and infarction areas in normal (50 cases) and pathological (100 cases) cases [63, 64]. The images were acquired roughly 10 min after the injection of a gadolinium-based contrast agent. The dataset is owned by the University Hospital of Dijon (France), but it is freely available.

### ToothFairy

Placing dental implant can become complex when the implant hits the inferior alveolar nerve. The *ToothFairy* dataset contains cone-beam computed tomography (CBCT) images and was released for a segmentation challenge in 2023 [90]. It extends the previous datasets (i.e. [98]) and comprises 443 dental scans with a voxel size of 0.3 mm^3^ yielding volumes with shapes ranging from (148, 265, 312) to (169, 342, 370) across the Z, Y, and X axes, respectively. The dataset includes 2D sparse annotations for all 443 vol, while only a subset of 153 vol contains detailed 3D voxel-level annotations. A team of five experienced surgeons delivered the ground truth [99, 100]. Additionally, a test set of 50 CBCT with a voxel size of 0.4 mm^3^ is provided for evaluation.

### HEad and neCK TumOR segmentation and outcome prediction (HECKTOR)

The training set of the *HECKTOR* challenge comprises 524 PET-CT volumes from seven hospitals with manual primary tumor and metastatic lymph nodes contours [73]. The data originates from FDG-PET and low-dose non-contrast-enhanced CT images of the head and neck region of subjects suffering from oropharyngeal cancer. The training set of the this challenge is provided to *MedShapeNet*.

### autoPET

Similar to *TotalSegmentor*, whole-body segmentations are extracted from the PET-CT dataset provided by the autoPET challenge [51], using an semi-supervised segmentation network [43]. The dataset comes from cancer patients and includes manual masks of tumor lesions.

### Calgary-campinas (CC)

This dataset provides high-quality anatomical data with 1 mm^3^ voxels from T1-weighted MRIs of 359 healthy subjects on scanners from three different vendors (GE, Philips, Siemens) at field strengths of 1.5 and 3 T [58]. The subjects vary in age and gender (176 M: 183 F, 53.5 ± 7.8 years, min: 18 years, max: 80 years). Probabilistic brain masks resulted from eight automated brain segmentation algorithms by simultaneous truth and performance level estimation (STAPLE) [101]. The quality of the masks was validated against 12 manual brain segmentations. Scientists investigate brain extraction models [102], domain shift and adaptation in brain MRI [103], as well as MRI reconstruction [104] using the CC dataset.

### Abdominal multi-organ benchmark for segmentation (AMOS)

The AMOS data includes 500 CTs and 100 MRIs from a variety of scanners and locations [48]. It provides expert segmentations of 15 abdominal organs: spleen, right kidney, left kidney, gallbladder, esophagus, liver, stomach, aorta, inferior vena cava, pancreas, right adrenal gland, left adrenal gland, duodenum, bladder, and prostate/uterus. Patients with abdominal tumors or other abnormalities delivered the images.

### AbdomenCT-1K and fast and low-resource abdominal organ segmentation (FLARE)

This dataset includes more than 1,000 CTs and manually generated masks of the liver, kidney, spleen, and pancreas [47]. A subset of the dataset was used in the [?] challenge, which provides expert segmentations of 13 abdomen organs the right and left kidney, stomach, gallbladder, esophagus, aorta, inferior vena cava, right adrenal gland, left adrenal gland, and duodenum [66] some of the CT scans are acquired from cancer patients.

### Ischemic stroke lesion segmentation (ISLES)

The *ISLES* challenge [75] provides 250 brain MRIs with binary masks depicting stroke infarctions. The dataset encompasses diverse brain lesions in terms of volume, location, and stroke pattern. Masks are generated by manually refining automatic segmentations from a 3D UNet [105].

### Synthetic anatomical shapes and shape augmentation

In addition to real anatomical shapes, we also provide synthetic shapes generated by generative adversarial net-works (GANs) [106]. For instance, we generate synthetic tumors for 27,390 real brains (Figure 3). Besides GANs, synthetic shapes can also be generated by registering two shapes and warping them to each other’s spaces [107]. This registration-based shape augmentation methods were used in the winning solutions of both the *AutoImplant I* and *AutoImplant II* challenges [26, 28].

### Medical instruments

In addition to anatomical shapes, *MedShapeNet* also provides 3D models of medical instruments [46], such as drill bits, scalpels, and chisels (Figure 4). We process the structured-light 3D scans using proprietary software (Ultrascan 2.0.0.7, Artec Studio 17 Professional) to remove noise. These models could help develop surgical tool tracking methods in mixed reality for medical education and research [32]. Realistic and accurate virtual surgical planning is performed in AR or VR [108], which improves the surgical outcome [109].

### Digital body preservation repository

These 3D models were captured from anatomical specimens using the handheld, high-resolution (accuracy 0.05 mm) structured-light surface scanner (Space Spider) and processed by the Studio 15 software (Artec 3D LUX, Luxembourg, Luxembourg) [62].

### Pathological shapes

To increase the variability of the shape collections, *MedShapeNet* contains not only normal/healthy anatomical shapes, such as the kidneys from *TotalSegmentor* and the brains from *HCP*, but also pathological ones, which are derived from patients diagnosed with a specific pathological condition, such as tumor (liver, kidney, etc.) and CUD (SUDMEX CONN, Table 2). Figure 3 shows the tumorous kidneys, brains, livers and head & neck, as well as diseased coronary arteries from different sources. We also use generative adversarial networks (GANs) to generate synthetic brain tumors, as shown in Figure 3.

## Annotation and example use cases

In *MedShapeNet*, *pairedness* is defined as having two composites i.e., the anatomical shapes and the metadata originating from the same subject, with one serving as input and the other as the ground truth. For instance, a 3D shape in *MedShapeNet* is paired with its anatomical category, such as ‘liver’, ‘heart’, ‘kidney’, and ‘lung’, which can be used for anatomical shape classification and retrieval. The metadata from DICOM or medical reports provides precise information about the source images, the patients (including attributes such as gender, age, body weight) as well as the diagnosis, and can deliver a variety of annotations. Synthetic shapes are distinguished from those obtained from real imaging data by the ‘synthetic’ label.

### Benchmarks derived from *MedShapeNet*

**Figure 6:**
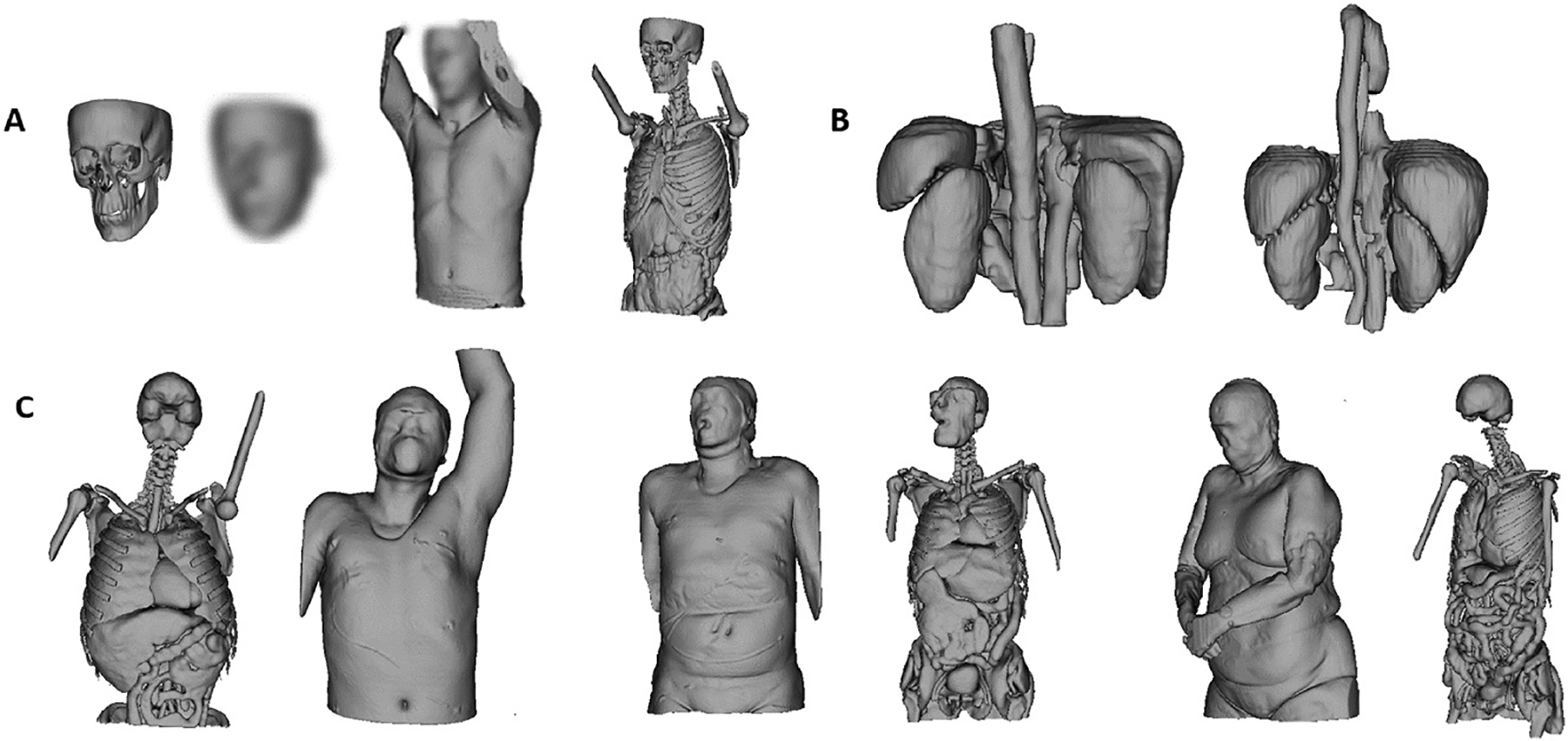
Examples of paired anatomical shapes in *MedShapeNet*. (A) Paired skins, muscles, fat, different tissues, organs and bones. (B) Paired abdominal anatomies, including liver, spleen, pancreas, right kidney, left kidney, stomach, gallbladder, esophagus, aorta, inferior vena cava, right adrenal gland, left adrenal gland, and duodenum. (C) Paired internal anatomies and body surfaces. For anonymity, the faces are blurred.

From *MedShapeNet* and its paired data, we can derive three types of benchmark datasets (Table 3):

- **Discriminative benchmarks** are comprised of 3D shapes and the corresponding anatomical categories and diagnosis. They can be used to train a classifier to discriminate 3D shapes (e.g., healthy, cancerous) based on shape-related features.
- **Reconstructive benchmarks** are composed of anatomical shapes derived from whole-body segmentations. They can be used in shape reconstruction tasks. For example, by training on paired skull-face shapes (Figure 6(A)), we can reconstruct human faces from the skulls automatically. We can also estimate an individual’s body composition, such as fat percentage or muscle distribution from the body surface [110, 111], by regressing on paired skin-fat or skin-muscle data (Figure 6(C)), or create a missing organ from its surrounding anatomies [30].
- **Variational benchmarks** are usually used for conditional reconstruction of 3D anatomical shapes. In addition to the geometric constraints imposed by the input shape, new reconstructions are expected to satisfy an additional attribute, such as age, gender or pathology. For example, it is possible to reconstruct multiple faces of different ages from the same skull, by introducing *age* as a constraint during supervised training. Similarly, a pathological condition, such as tumor, can be imposed on healthy anatomies, or the morphological changes of an anatomy during disease progression can be modeled [112]. Variational auto-encoder (VAE) [113] and GANs are commonly used for such conditional reconstruction tasks.

### Example use cases of *MedShapeNet*

**Figure 7:**
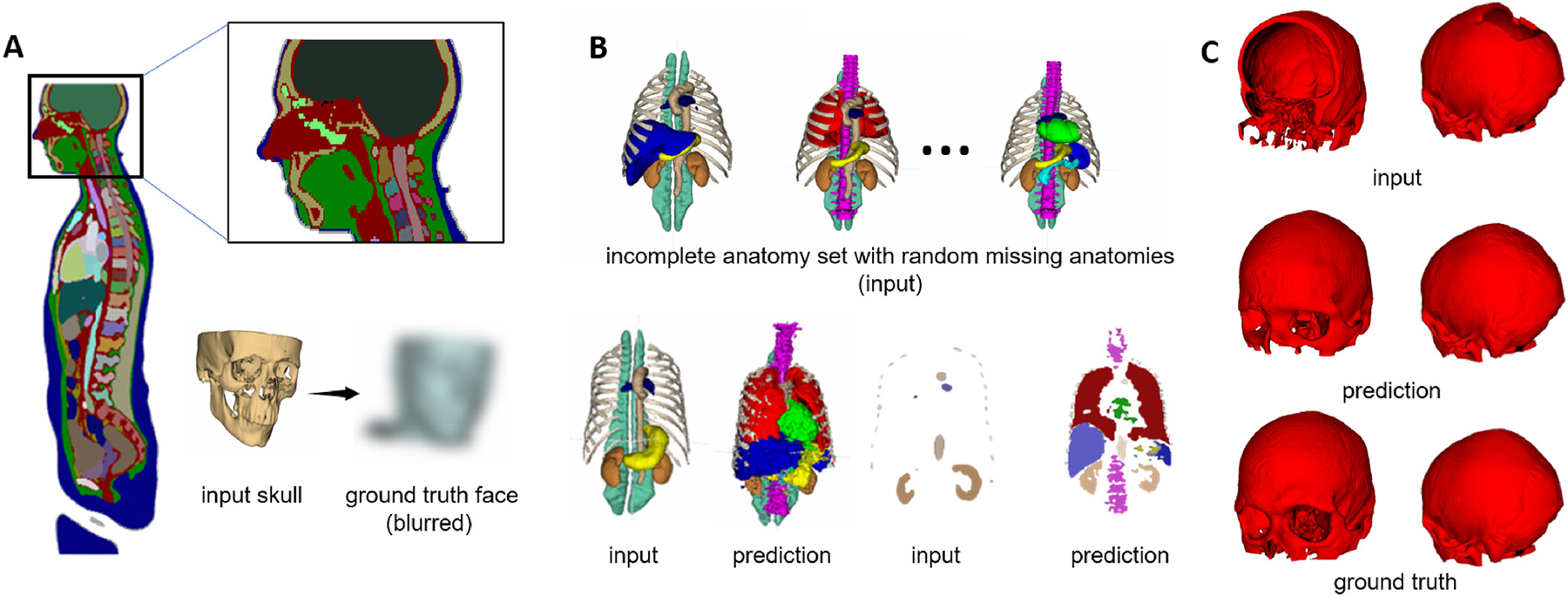
Benchmarks for various vision applications can be derived from *MedShapeNet*, such as (A) forensic facial reconstruction, (B) anatomical shape reconstruction, and (C) skull reconstruction.

**Figure 8:**
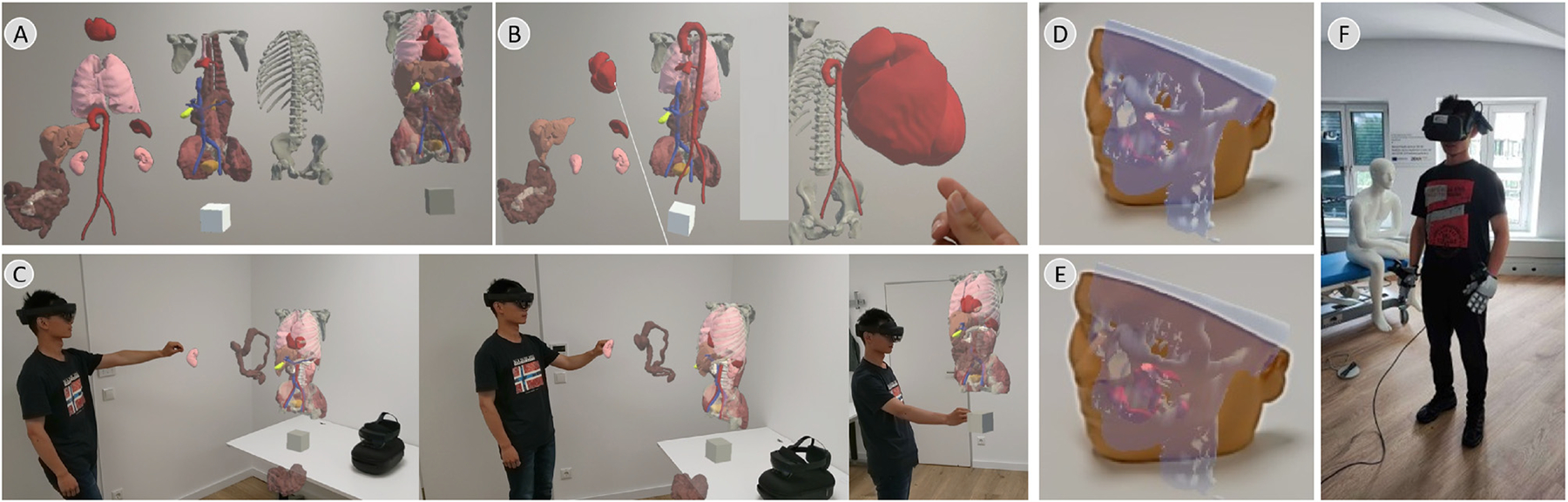
A use case of *MedShapeNet* in AR- and VR-based anatomy education. (A) A whole-body model from *MedShapeNet* dissembled into individual anatomies. (B, C) anatomy manipulation in first- and third-person views. (D, E) A 3D-printed facial phantom and the corresponding skull and tumors. (F) Using haptic VR gloves to interact with the 3D anatomical models in the virtual environment.

To illustrate the unique value of *MedShapeNet*, we describe five real-world use cases and show how *MedShapeNet* is used to solve vision/medical problems:

- **Tumor classification** of brain lesions is usually based on gray-scale MRIs [114, 115]. In this use case, we train a convolutional neural network (CNN)-based classifier to discriminate between tumorous and healthy brain shapes. The classifier has shown good convergence and generalizability. Similar results are observed for the classification of brain shapes from males and females, in line with existing studies [116].
- **Facial reconstruction** is a common practice in archeology, anthropology and forensic science, where the objective is to recreate the facial appearances of historical figures, ancient humans or victims from their skeletal remains [117]. Orthognathic surgery also employs this technology to predict postoperative outcomes [118]. Nevertheless, in addition to the skull, the facial appearance is also significantly influenced by factors such as the quantity and distribution of facial fat and muscles [119], making facial reconstruction a highly ill-posed problem in terms of the skull-face relationship (Figure 7(A)).
- **Skull reconstruction** aims to rebuild missing parts of the skull bones around the facial area or the cranium (Figure 7(C)), where both voxel grids [26, 28, 120] and point clouds [121, 122] have been used to represent the skull data.
- **Anatomy completion** investigates the feasibility of automatically generating whole-body segmentations given only sparse manual annotations. The generated segmentations can subsequently be used as pseudo labels to train a whole-body segmentation network [30]. Figure 7(B) provides an example input and the corresponding reconstruction results.
- **Extended reality (XR)** combines real and virtual worlds. *MedShapeNet* can also benefit a variety of XR (AR/MR/VR) applications that require 3D anatomical models [123], such as virtual anatomy education [124]. Figure 8(A) shows a whole-body model using the *Microsoft HoloLens* AR glasses. The user can dissemble individual anatomies, move them, zoom in and out, and rotate the structures (Figure 8(B) and (C)). Furthermore, if necessary, we can 3D print the models (Figure 8(D) and (E)). Users can also wear VR gloves (Figure 8(F)) to receive haptic feedback while interacting with the 3D anatomies in VR [125].

## *MedShapeNet* interface

Two interfaces are created for *MedShapeNet*, including a web-based interface that provides access to the original high-resolution shape data, and a Python API that enables users to interact with the shape data via Python.

### Web-based interface

A user-centric, intuitive web-based interface^3^ has been developed to provide convenient access to the shape data within *MedShapeNet*, which allows users to search, retrieve, and view individual shapes. Shapes can be retrieved using queries related to anatomical category such as *heart*’, *brain*, *hip*, *liver*, or pathologies like *tumor*. A dedicated GitHub page^4^ has also been established to manage shape contribution and removal (in case of inaccurate shapes), feature requests and the open-sourcing of applications based on *MedShapeNet*.

### *MedShapeNetCore* and python API

We have also developed a Python API that facilitates the integration of the dataset into Python-centric workflows for computer vision and machine learning. This API grants access to a standardized subset of the original *MedShapeNet* dataset, referred to as *MedShapeNetCore*, which has been specifically curated for the efficient and reliable benchmarking of various vision algorithms. *MedShapeNetCore* differs from the original dataset in aspects:

- **Resolution.** The original 3D models are prohibitively high resolution to be used directly by vision algorithms.^5^ In contrast, *MedShapeNetCore* contains considerably more lightweight 3D models and lower resolution images, similar to those in *ShapeNet* [13] and *MedMNIST* [126].
- **Quality.** The 3D models in *MedShapeNetCore* are water-tight and the quality of each individual model has been meticulously verified through manual inspection.
- **Annotation.** *MedShapeNetCore* is more densely annotated, expanding its applicability to tasks such as shape part segmentation [127] and anatomical symmetry plane estimation.

The 3D shapes are stored in the standard formats for geometric data structures, i.e., NIfTI (.nii) for voxel grids, stereolithography (.stl) for meshes and Polygon File Format (.ply) for point clouds, facilitating fast shape preview via existing softwares. The Python API facilitates the loading of these shape data into standard *Numpy* arrays, ensuring a seamless transformation into tensor representations compatible with various deep learning frameworks, including but not limited to *PyTorch*, *MONAI*, and *Tensor-Flow*. The light-weight nature of these data expedites the process of developing new medical vision algorithms or evaluating existing ones, while maintaining a low computational overhead. The ongoing efforts in the development of the Python API include integrating PyTorch3D [128] to leverage its sophisticated 3D operators, establishing predefined benchmarks tailored for various vision and medical applications, and incorporating pre-trained models and shape processing algorithms.

## Discussion

High-quality, annotated datasets are valuable assets for data-driven research. We created MedShapeNet as an open, ongoing effort and requires continuous contributions from these communities. We believe that *MedShapeNet* holds the potential to make significant contributions to research in medical imaging and computer vision. It could impact the practice of medical data curation and sharing, as well as the development of data-driven methods for medical applications.

Compared to vision datasets, large medical datasets are more difficult to curate due to the sensitive, distributed, and scarce nature of medical images. Therefore, the medical imaging community has recently started catching up with the development of vision algorithms that can exploit large datasets, with more and more medical researchers becoming open to data-sharing. Thus, *MedShapeNet* provides a versatile dataset that both vision and medical researchers are accustomed to.

To avoid potentially harmful societal impact, computer vision research involving human-derived data should be conducted with care. We designed MedShapeNet specifically for research, and the researchers shall follow ethical guidelines throughout methodology development and experimental design. For example, publicly sharing neuroimaging data bears high privacy risks and needs regulation, since they contain patients’ facial profiles [129]. For instance, Schwarz et al. recently identified participants in a clinical trial comparing their faces reconstructed from MRI with photographs on social media [130]. Therefore, besides removing patients’ meta informationfrom DICOM tags, defacing is also commonly practiced [131]. However, we have shown that machine learning can reconstruct skulls even when they are damaged or parts of the bones are missing. Another double-edged use case of *MedShapeNet* is training machine learning to detect substance (drug or alcohol) addiction or other diseases e.g., fetal alcohol syndrome (FAS), based on facial characteristics [132]. Furthermore, since *MedShapeNet* preserves the correspondence between the shapes and patients’ meta information, such as age, race, gender, medical history, etc., which facilitates the learning of some controversial mapping relationships. Potentially, the ethnic identity or medical history is predicted from a person’s skull or facial profiles [133]. It is therefore the responsibility of the researchers to weigh the social benefits against the potential negative societal impacts while developing models using *MedShapeNet*.

For future developments, we will primarily focus on the following aspects:

- Incorporating a greater number of datasets and metadata as well as pathological shapes, particularly those pertaining to rare diseases.
- Advocating for *MedShapeNet* through presentations at conferences, symposia, and seminars, as well as organizing hackweeks, workshops, and challenges.
- Establishing additional benchmarks and use cases.
- Enhancing the web and Python interfaces.

## Conclusions

In this white paper, we have introduced the initial efforts for MedShapeNet. We (1) formed a community for data contribution; (2) derived open-source benchmark datasets for several use cases; (3) constructed interfaces to search to download the shape data and its paired information; (4) brought up several interesting shape-related research topics; and (5) discussed the relevance of ethical guidelines and precautions for privacy of medical data.

## Figures and Tables

**Table 1:** A non-inclusive list of organizations & events featuring shape and computer vision methods for medical applications.

Sources	Description	Category
Zuse Institute Berlin (ZIB)	Shape-informed medical image segmentation and shape priors in medical imaging	Research group
ShapeMI	Shape processing/analysis/learning in medical imaging	MICCAI workshop
SIG	Shape modeling and analysis in medical imaging	MICCAI special interest group (SIG)
AutoImplant I, II	Skull shape reconstruction and completion	MICCAI challenge
WiSh	Women in shape analysis, shape modeling	Professional organization
STACOM	Statistical atlases and computational models of the heart	MICCAI workshop
SAMIA	Shape analysis in medical image analysis	Book
CIBC	Image and geometric analysis	Research group
GeoMedIA	Geometric deep learning in medical image analysis	MICCAI-endorsed workshop
IEEE TMI	Geometric deep learning in medical imaging	Journal special issue
PMLR	Geometric deep learning in medical image analysis	Proceedings
Elsevier	Riemannian geometric statistics in medical image analysis	Book
Springer	Geometric methods in bio-medical image processing	Proceedings
MCV	Workshop on medical computer vision	CVPR workshop
MCV 2010–2016	Workshop on medical computer vision	MICCAI workshop
MeshMed	Workshop on mesh processing in medical image analysis	MICCAI workshop

**Table 2:** The sources segmentation datasets (ordered alphabetically).

Sources	Description	Dataset license
AbdomenAtlas [44]	25 organs and seven types of tumor	–
AbdomenCT-1K [47]	Abdomen organs	CC BY 4.0
AMOS [48]	Abdominal multi organs in CT and MRI	CC BY 4.0
ASOCA [49, 50]	Normal and diseased coronary arteries	–
autoPET [43, 51–53]	Whole-body segmentations	CC BY 4.0
AVT [54]	Aortic vessel trees	CC BY 4.0
BraTS [55–57]	Brain tumor segmentation	–
Calgary-campinas [58]	Brain structure segmentations	–
Crossmoda [59, 60]	Brain tumor and cochlea segmentation	CC BY 4.0
CT-ORG [61]	Multiple organ segmentation	CC0 1.0
Digital body preservation [62]	3D scans of anatomical specimens	–
EMIDEC [63, 64]	Normal and pathological (infarction) myocardium	CC BY NC SA 4.0
Facial models [65]	Facial models for augmented reality	CC BY 4.0
FLARE [47, 66–68]	13 abdomen organs	–
GLISRT [69–71]	Brain structures	TCIA restricted
HCP [72]	Paired brain-skull extracted from MRIs	Data use terms
HECKTOR [73, 74]	Head and neck tumor segmentation	–
ISLES22 [75]	Ischemic stroke lesion segmentation	CC-BY-4.0
KiTS21 [76]	Kidney and kidney tumor segmentation	MIT
LiTS [77]	Liver tumor segmentation	–
LNDb [78, 79]	Lung nodules	CC BY NC ND 4.0
LUMIERE [80]	Longitudinal glioblastoma	CC BY NC
MUG500+ [81]	Healthy and craniotomy CT skulls	CC BY 4.0
MRI GBM [82]	Brain and GBM extracted from MRIs	CC BY 4.0
PROMISE [83]	Prostate MRI segmentation	–
PulmonaryTree [84]	Pulmonary airways, arteries and veins	CC BY 4.0
SkullBreak [85]	Complete and artificially defected skulls	CC BY 4.0
SkullFix [85]	Complete and artificially defected skulls	CC BY 4.0
SUDMEX CONN [86]	Healthy and (cocaine use disorder) CUD brains	CC0
TCGA-GBM [57]	Glioblastoma	–
3D-COSI [46]	3D medical instrument models	CC BY 4.0
3DTeethSeg [87, 88]	3D teeth scan segmentation	CC BY NC ND 4.0
ToothFairy [89, 90]	Inferior alveolar canal	CC BY SA
TotalSegmentator [42]	Various anatomical structures	CC BY 4.0
VerSe [91]	Large scale vertebrae segmentation	CC BY 4.0

**Table 3:** Instances of *MedShapeNet* benchmarks.

Discriminative benchmarks		Reconstructive benchmarks		Variational benchmarks	
Input (shape)	Ground truth (metadata)	Input (shape)	Ground truth (shape)	Input (shape + metadata)	Ground Truth (Shape)
Liver/kidney/brain	Tumor/healthy	Skull	Face	Face + AUD/CUD/AD/age	Face
Brain	AUD/CUD/AD/age	Ribs + spines	Torso organs	Brain + AUD/CUD/AD/age	Brain
Face	AUD/CUD/age/gender	Skin	Body fat/muscle/skeleton	–	–
3D shapes	Anatomical categories	Full skeleton	Skin	–	–

AD, Alzheimer’s disease; AUD, alcohol use disorder; CUD, cocaine use disorder.

## Data Availability

https://medshapenet.ikim.nrw/.
